# A Case Report of SMARCA4-Deficient Thoracic Sarcoma: A Rare and Aggressive Disease With a Grim Prognosis

**DOI:** 10.7759/cureus.39571

**Published:** 2023-05-27

**Authors:** Siham Lalaoui Rachidi, Nelly Firmin, Mohamed Elfadli, Ismail Essadi, Rhizlane Belbaraka

**Affiliations:** 1 Medical Oncology, Mohammed VI University Hospital, Marrakesh, MAR; 2 Medical Oncology, Montpellier Cancer Institute (ICM), Montpellier, FRA; 3 Medical Oncology, Ibn Sina Military Teaching Hospital Marrakesh, Marrakesh, MAR

**Keywords:** swi/snf, sarcoma, thoracic, smarca4, smarca4-dts, sarcomatoid carcinoma

## Abstract

SMARCA4-deficient thoracic sarcoma (DTS) is a rare malignancy defined by inactivating SMARCA4 mutations leading to protein loss. It was recently described as an aggressive disease with a dismal prognosis, mostly affecting young men with a history of heavy smoking. Histologically, SMARCA4-DTS is a poorly differentiated tumor with rhabdoid or epithelioid features that can be distinguished from other soft tissue, and thoracic sarcomas by a higher tumor mutation burden (TMB) and the presence of smoking signatures, including KRAS, STK11, and KEAP1 mutations. Currently, there is no approved treatment for SMARCA4-DTS, which is known to be chemo-resistant, but more recent studies have shown some effectiveness with immune checkpoint inhibitors.

We report the case of a 42-year-old man with a family history of cancer who was admitted to the hospital with acute respiratory distress and superior vena cava syndrome. He had been experiencing thoracic pain, dry cough, dyspnea, fatigue, and unintentional weight loss for a month. Imaging revealed multiple masses and lymph nodes in the chest, as well as pleural effusion. PET scan showed widespread metastases. A cervical lymph node biopsy confirmed the diagnosis of SMARCA4-deficient thoracic sarcoma. Unfortunately, his general condition did not allow an aggressive treatment. He was started on Pazopanib 800mg per day, but deteriorated rapidly and passed away.

This report highlights the aggressive nature and unfavorable prognosis associated with SMARCA4-deficient thoracic sarcoma. Accurate diagnosis of this entity can be challenging due to its unique marker expression and unfamiliar histological features. Currently, there are no established treatment strategies for this condition; however, recent studies have shown promising results with immune checkpoint inhibitors and targeted therapies. Further research is necessary to identify the most effective treatment approaches for SMARCA4-DTS.

## Introduction

SMARCA4-deficient thoracic sarcoma (DTS) is a newly reported entity caused by an inactivating mutation leading to the loss of SMARCA4 expression, one of the major components of the switch/sucrose-non-fermentable (SWI/SNF) chromatin remodeling complex [1]. Twenty percent of various malignancies from different sites, such as the uterine, gastrointestinal system, and lungs, have been shown to harbor this mutation [2]. It predominantly affects young men with a history of smoking [3]. Currently, there is no established therapy for this disease, and the prognosis is poor, with a median survival of only four to seven months [4].

Herein, we report the clinical course of a rapidly fatal SMARCA4-DTS in a young man, along with a review of the literature to provide further insight into the clinical, immunohistochemical, and genetic sequencing characteristics of these aggressive tumors.

## Case presentation

A 42-year-old man with no prior health problems was admitted to our hospital with acute respiratory distress and superior vena cava syndrome. Upon inquiry, we discovered that he had a family history of cancer on his mother’s side (his mother died of colon cancer, an unlabeled lung tumor in an aunt, and lymphoma in the grandfather). He was a current smoker with a history of 10 pack years.

**Figure 1:**
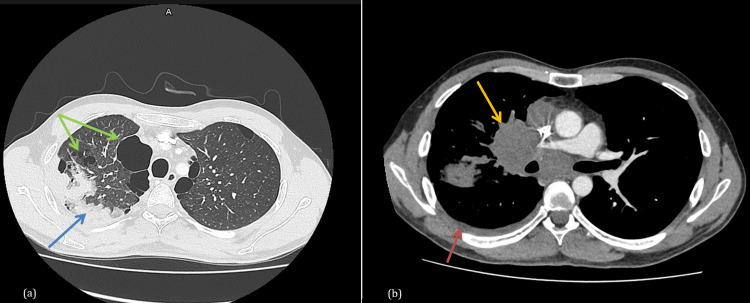
Computed tomography (CT) of the chest (a) Lung window showing: Multiple confluent intraparenchymal masses of the right upper lobe (blue arrow) associated with emphysema, bullous dystrophy, and diffuse bronchial syndrome (green arrows). (b) Mediastinal window showing voluminous mediastinal lymph nodes (yellow arrow), and pleural effusion on the right side (red arrow).

The patient had been experiencing thoracic pain, dry cough, dyspnea, fatigue, and unintentional weight loss for a month. A thoracic CT scan revealed multiple intraparenchymal masses, voluminous mediastinal lymph nodes, and pleural effusion on the right side, as well as radiological evidence of emphysema, bullous dystrophy, and diffuse bronchial syndrome (Figure 1).

He was admitted to the pulmonology department for further examination. A cervical lymph node biopsy showed lymphoid material with clusters of large epithelioid cells that had a large irregular nucleus and cytoplasm that was sometimes clarified. The mitoses were numerous, and there were some foci of necrosis. On immunohistochemistry, the tumor cells were positive for EMA, SALL4, SOX2, CD99, and CD30 (low) but negative for CK, CD20, CD45, CD3, CD2, ALK, Kit, BGR1, SOX10, CD56, CD1a, and PLAP. This morphological and phenotypic aspect is related to a SMARCA4-deficient thoracic sarcoma.

**Figure 2:**
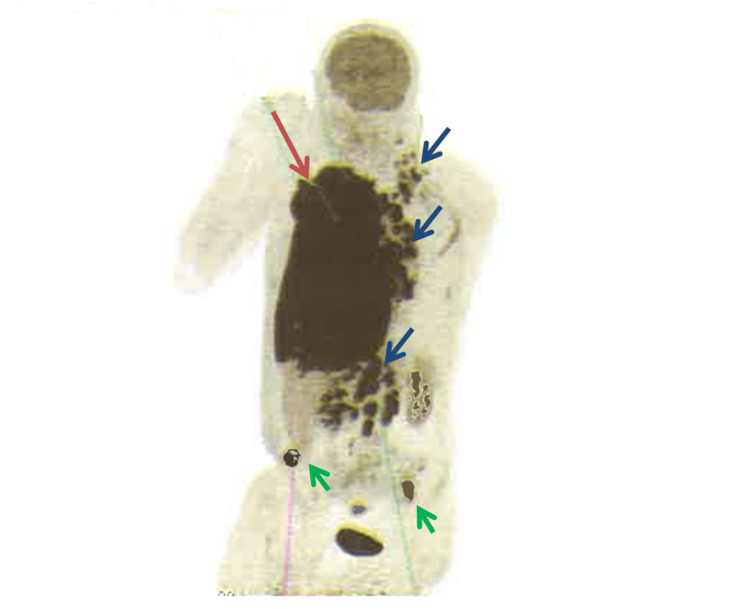
Whole body positron emission tomography PET/CT Hypermetabolic left supraclavicular, hilar, mediastinal, and subdiaphragmatic adenopathies (blue arrows). Intense hypermetabolism of the right pleural thickening associated with two bony hypermetabolic lesions were found in the iliac bone and the sacrum (green arrows), indicating a possible secondary location.

A PET scan showed hypermetabolic left cervical, bilateral supraclavicular, hilar, mediastinal, and subdiaphragmatic adenopathies. There was also intense hypermetabolism of a right pleural thickening, associated with bilateral pleural effusion. Additionally, two bony hypermetabolic lesions were found in the iliac bone and the sacrum, indicating a possible secondary location (Figure 2).

During his hospitalization, he presented with superior cava syndrome and received curative anticoagulation with stenting. He also had an increase in the inflammatory syndrome due to bronchial congestion, requiring broad-spectrum antibiotic therapy. He was referred to us to initiate oncological treatment.

Upon admission to our department, the patient had general deterioration with a performance status of 4. His vitals were good, and his oxygen blood level was 94% on 2 liters of oxygen. He had dyspnea while speaking, intercostal pulling, and thoracoabdominal rocking. There were also several bilateral cervical and supraclavicular lymph nodes, right facial edema, right hemithorax, and upper extremity edema associated with collateral venous circulation. Pulmonary auscultation showed a right effusion syndrome of moderate size. The rest of the examination was unremarkable.

Regrettably, his overall health did not allow an intensive approach. He was started on Pazopanib 800mg per day; however, his general state declined rapidly while under our care, and he passed away shortly thereafter.

## Discussion

The SMARCA4-encoded protein BRG1 is one of the major components of the SWI/SNF chromatin remodeling complex, which is involved in regulating transcription, promoting cell differentiation, and repairing DNA [5]. The loss of expression and inactivating mutations in multiple components of this complex have been linked to the development of cancer and can be observed in up to 20% of different types of tumors [6].

SMARCA4 is considered a tumor suppressor gene, and its implication in carcinogenesis has emerged only in the previous decade, following the initial description of SMARCA4 deficiency in pediatric malignant rhabdoid tumors (MRT) and subsequently its identification in small cell carcinomas of the ovary, hypercalcemic type (SCCOHT), and other tumor types, such as lung adenocarcinomas [3,7].

SMARCA4-deficient thoracic sarcoma is a newly proposed type of malignancy that is characterized by rapidly growing masses in the lungs, mediastinum, and pleura. It is a rare and aggressive cancer with distinctive histomorphological and molecular features that was first described in 2015 by Le Loarer et al. [1]. The phenotypic expression profile is comparable to SCCOHT and MRT and different from non-small cell lung cancer (NSCLC), indicating a distinct kind of thoracic sarcoma [4]. The pathologist community acknowledged this entity in the fifth edition of the World Health Organization's (WHO) classification of thoracic tumors. However, they changed its name from SMARCA4-deficient thoracic sarcomas to SMARCA4-deficient undifferentiated tumors (UT) [8].

According to reported cases in the existing medical literature, the majority of patients are male with a history of heavy smoking. Interestingly, the age range of these patients is quite wide, encompassing those who fall within the typical age range for NSCLC (>50-80 years old) as well as those who are unusually young (between 30 and 50 years old) [3,4]. Clinically, it manifests with nonspecific upper and lower respiratory symptoms caused by extremely large and rapidly progressive masses involving the lung, mediastinum, and pleura [9].

Most patients are diagnosed at stage IV, and the pattern of metastasis may resemble that of pulmonary carcinomas (with involvement of the lymph nodes, adrenal glands, and bones), but it also has multiple distinctive features such as the absence of brain metastases and large peritoneal masses [4,7].

Histologically, SMARCA4-deficient UT can appear entirely sarcomatoid or exhibit both rhabdoid and epithelioid features. Uniform, round cell structure with mild pleomorphism and large nucleoli, accompanied by extensive necrosis and high mitotic activity are all characteristics of sarcomatoid components [7].

Immunohistochemistry studies often reveal the absence of SMARCA2, keratin, and claudin-4 expression but detect the presence of vimentin and synaptophysin expression. TTF1 and P40 are weak and focally expressed, while stem cell markers, including Sal-like protein 4 (SALL4) and CD34, as well as other markers like CD99 and SMARCB1, are also expressed in certain cases. The Ki-67 proliferation rate is consistently high at around 70% [10].

Molecular testing often identifies a high tumor mutation load, including TP53 and SMARCA4 alterations in addition to smoking-associated NSCLC mutations, such as STK11, KEAP1, and KRAS, including KRAS G12C, which are usually absent in sarcomas [2].

Rekhtman and colleagues confirm previous findings indicating the variability of genomic mechanisms responsible for SMARCA4 inactivation. While large chromosomal losses or deletions are only occasionally observed, these mechanisms are primarily truncating mutations combined with loss of heterozygosity (LOH) [3]. Second, they found that in most cases, LOH is copy-neutral (i.e., involves duplication of the mutated allele) and therefore cannot be detected by fluorescence in situ hybridization (FISH), unlike in SMARCB1-deficient neoplasms. Thus, FISH has a limited role in assessing SMARCA4-deficient UT. An accurate diagnosis is complex and requires obtaining an adequate tissue sample that must be reviewed by an expert pathologist [11].

In some cases, cancer may be identified as an undifferentiated carcinoma or a tumor of unknown primary, and the presence of neuroendocrine markers and high Ki-67 may lead to misdiagnosis.

Treatment strategies for these tumors have not been established. Previous studies have shown that Adriamycin and ifosfamide have limited benefit in SMARCA4-deficient UT, which are known to be chemo-resistant, but more recent studies have demonstrated some effectiveness with immune checkpoints such as nivolumab and pembrolizumab [2,11,12]. Although exceptional responders were identified, immune checkpoint inhibitors have shown limited efficacy in SMARCA4-deficient UT patients with observed objective response rates consistently below 20%, despite promising preclinical data [13].

Therapeutic approaches to tumors with SMARCA4 mutations and other SWI/SNF complex alterations are currently the subject of active research, in particular enhancers of zeste homolog 2 (EZH2) inhibitors and cyclin-dependent kinase (CDK) 4/6 inhibitors [14-16]. It is worth mentioning that the finding of high tumor mutation burden (TMB) suggests that immune checkpoint inhibitors may be a promising option to consider for SMARCA4-deficient UT [17].

Overall, the identification of treatment approaches for SMARCA4-deficient UT can be facilitated by a better understanding of the pathology and molecular characteristics of these tumors. Although recent cases reported a prolonged response to immunotherapy, the prognosis is typically grim, with overall survival ranging from weeks to months [4,11,18,19].

## Conclusions

Our report confirms the aggressive behavior and poor prognosis of SMARCA4-deficient UT. The peculiar marker expression and lack of familiarity with the histologic features of this recently described entity can present a major diagnostic challenge. Thorough immunohistochemical analyses plus a smoking history are required to diagnose SMARCA4-deficient thoracic sarcoma. Despite the lack of established treatment strategies, recent studies have shown some effectiveness with immune checkpoint inhibitors and other targeted therapies. Further research is needed to identify optimal treatment approaches for SMARCA4-deficient UT, which can be facilitated by a better understanding of the pathology and molecular characteristics of these tumors.
